# The renin–angiotensin–aldosterone system: Role in pathogenesis and potential therapeutic target in COVID‐19

**DOI:** 10.1002/prp2.623

**Published:** 2020-07-13

**Authors:** Cássia L. Braga, Rodrigo P. Silva‐Aguiar, Denise Battaglini, Diogo B. Peruchetti, Chiara Robba, Paolo Pelosi, Patricia R. M. Rocco, Celso Caruso‐Neves, Pedro L. Silva

**Affiliations:** ^1^ Laboratory of Pulmonary Investigation Carlos Chagas Filho Institute of Biophysics Federal University of Rio de Janeiro Rio de Janeiro Brazil; ^2^ Laboratory of Biochemistry and Cell Signalling Carlos Chagas Filho Institute of Biophysics Federal University of Rio de Janeiro Rio de Janeiro Brazil; ^3^ Anesthesia and Intensive Care Ospedale Policlinico San Martino IRCCS for Oncology and Neuroscience Genoa Italy; ^4^ Department of Surgical Sciences and Integrated Diagnostic (DISC) University of Genoa Genoa Italy; ^5^ National Institute of Science and Technology for Regenerative Medicine Rio de Janeiro Brazil; ^6^ Rio de Janeiro Innovation Network in Nanosystems for Health‐NanoSAÚDE/FAPERJ Rio de Janeiro Brazil; ^7^ COVID‐19 Virus Network Ministry of Science and Technology, Innovation and Communication Rio de Janeiro Brazil

**Keywords:** angiotensin‐converting enzyme, COVID‐19, inflammation, SARS‐CoV‐2, therapy

## Abstract

Coronavirus disease 2019 (COVID‐19), caused by the SARS‐CoV‐2 novel coronavirus, has spread worldwide causing high fatality rates. Neither a vaccine nor specific therapeutic approaches are available, hindering the fight against this disease and making better understanding of its pathogenesis essential. Despite similarities between SARS‐CoV‐2 and SARS‐CoV, the former has unique characteristics which represent a great challenge to physicians. The mechanism of COVID‐19 infection and pathogenesis is still poorly understood. In the present review, we highlight possible pathways involved in the pathogenesis of COVID‐19 and potential therapeutic targets, focusing on the role of the renin–angiotensin–aldosterone system.

**Abbreviations**
COVID‐19: coronavirus disease 2019
ICU: intensive care unit
RAAS: renin–angiotensin–aldosterone system
S: spike
WHO: World Health Organization

## 1 INTRODUCTION

In December 2019, an atypical viral pneumonia caused by a novel coronavirus was identified in Wuhan, China (https://www.who.int/). Within months, the disease, later named coronavirus disease 2019 (COVID‐19) by the World Health Organization (WHO), had spread worldwide and become a global health emergency.^1^ According to WHO, as of June 09, 2020, the number of confirmed cases was over 7,039,918 and the number of deaths more than 404 396. (https://www.who.int/). United Kingdom, Spain, and Italy followed by France, have the highest number of cases in Europe, while the United States represents the epicenter of the disease in the American continent. Some countries, such as China, Germany, and Denmark, are exhibiting a progressive decline in cases, with hopes that the pandemic has not only peaked but also been controlled in these territories (https://covid19.who.int).

The median age of infected individuals who need hospitalization ranges from 49 to 56; however, patients who need intensive care unit (ICU) care have been significantly older, with a median age of approximately 66 years.^2^, ^3^, ^4^ Moreover, individuals with chronic comorbidities such as hypertension and diabetes are at the highest risk of poor outcomes when infected.^5^, ^6^ The clinical picture of COVID‐19 ranges from asymptomatic to severe respiratory failure. The main symptoms are fever, fatigue, and cough; patients can be classified as mild, severe, and critical according to clinical presentation.^7^ Different pathophysiological pathways have been identified and explored, but there is no clear evidence of protective or risk factors for SARS‐CoV‐2 infection. In the present review, we highlight possible pathways involved in the pathogenesis of COVID‐19, with focus on the role of the renin–angiotensin–aldosterone system (RAAS).

## 2 ACE2 IN SARS‐COV‐2 INFECTION

**FIGURE 1:**
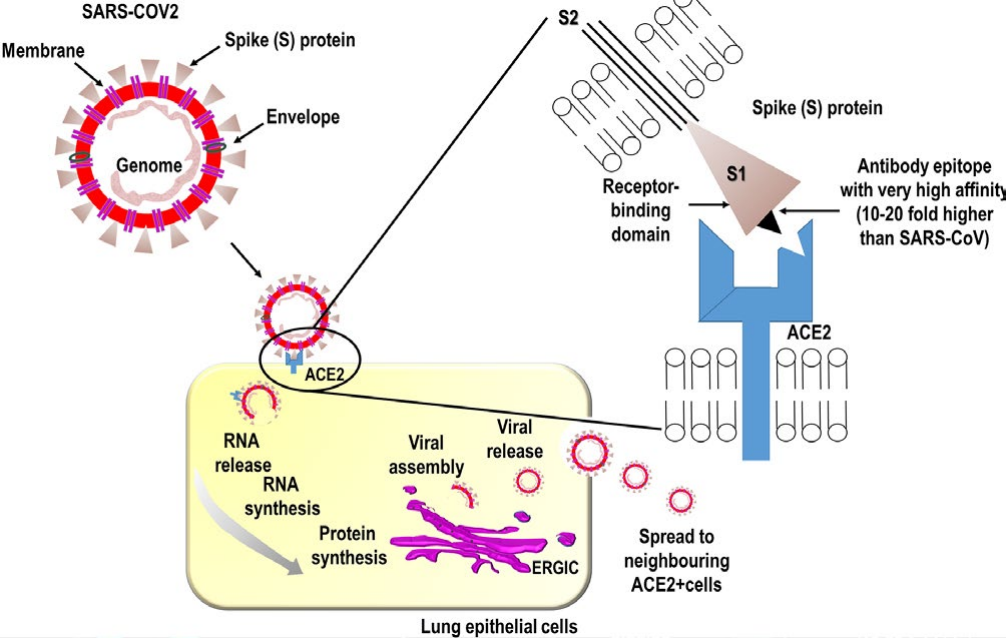
Structural proteins of SARS‐COV‐2: spike (S), envelope (E), and matrix (M), as well as nucleocapsid (N) proteins 3‐5. The S protein is divided into two subunits, S1 and S2. The S1 domain attaches to cells through angiotensin‐converting enzyme 2 (ACE2). The resulting virus‐ACE2 complex is translocated into the cell and a host protease cleaves the S2 domain, which releases the viral genome into the cytoplasm. In the cytoplasm, the viral genome is translated into replicase polyproteins that drive RNA synthesis and replication. Virus structural and nonstructural proteins are then synthesized using intracellular machinery. These proteins bud into the endoplasmic reticulum‐Golgi intermediate compartment (ERGIC); new viral particles are then assembled and released to infect new target cells

A key structural component of all coronaviruses is the envelope‐anchored spike (S) protein, which enables the virus to bind to receptors on the host cell (Figure 1).^8^, ^9^ According to Zhou et al, SARS‐CoV‐2 uses the angiotensin‐converting enzyme 2 (ACE2) receptor to invade and infect cells.^10^ Hoffmann et al further suggested that a host cell protease is necessary to allow virus fusion.^11^

ACE2 is a type I integral monocarboxypeptidase with 46% homology to ACE protein sequence.^12^, ^13^ Structurally, ACE2 has a catalytical metalloprotease domain, a signal peptide, and a transmembrane domain.^12^ Its extracellular catalytic domain contains a substrate binding region and zinc‐binding site critical for its activity. Usually, its cleavage site is preceded by a X‐Pro or Pro‐X‐Pro motif.^14^ ACE2 cleavages Ang II at C‐terminal domain removing phenylalanine (^7^Pro‐^8^Phe) forming angiotensin (1‐7) (Ang(1‐7)).

ACE2 is highly expressed in lung epithelial and endothelial cells, which explains the primary occurrence of respiratory system dysfunction during COVID‐19 infection.^15^ Among lung cells, it has been observed that virus‐related genes were more likely expressed in type 2 lung epithelial cells, which may explain the severe alveolar damage seen after infection. Nevertheless, ACE2 is also expressed in the kidney, heart, tongue, ileum, and esophagus, thus explaining the presence of nonrespiratory symptoms.^16^, ^17^

As noted above, binding of SARS‐CoV‐2 to ACE2 is mediated by the S protein.^11^, ^18^ The SARS‐CoV‐2 S protein has approximately 76% homology with that of SARS‐CoV, the virus which caused the 2002‐2004 severe acute respiratory syndrome outbreak.^19^ He et al observed that a mutation leading to substitution of arginine residue 44 of the SARS‐CoV S protein by alanine (R44A) abolished its binding to ACE2.^20^ Structural data have demonstrated that the SARS‐CoV‐2 receptor binding domain (RBD), located in the S1 domain of the spike protein, interacts with the catalytic domain of ACE2.^21^, ^22^, ^23^, ^24^

Importantly, SARS‐CoV‐2 carries mutation in the spike protein RBD that could confer higher affinity for ACE2 when compared to the SARS‐CoV spike protein, including Val404 to Lys307 and Arg426 to Asn439, due to salt bridge and van der Waals contact, respectively, as shown by cryo‐EM interface comparison.^22^, ^24^ This could explain, at least in part, the higher transmissibility of SARS‐CoV‐2.^21^, ^22^, ^23^

Upon engagement of ACE2, SARS‐CoV‐2 S protein is primed by the transmembrane serine protease 2 (TMPRSS2) in two distinct subunits, S1 and S2, a step essential for efficient virus replication.^11^ Importantly, despite the suggestion that SARS‐CoV could fuse with the host plasma memrane after ACE2 binding,^25^ it has been demonstrated that viral entry is also mediated by clathrin‐independent and caveolin‐independent endocytosis,^26^ involved in lipid raft formation crucial for SARS‐CoV internalization.^26^ However, the molecular mechanism involved in SARS‐CoV‐2 infection has not been completely determined. An important clue comes from the observation that endosomal alkalization induced by ammonium chloride inhibited SARS‐CoV‐2 replication.^11^ Ou et al,^19^ using in vitro infection of HEK293/hACE2 cells (expressing ACE2) with SARS‐CoV‐2 S pseudovirions, observed that virus infection required the cell surface endocytosis. In agreement with this finding, it has been observed that drugs such as chloroquine, which halt the progression from early endosome to lysosome, decrease the viral replication rate.^27^ These observations have sparked great interest in the potential utility of these drugs for treatment of COVID‐19, although no scientific evidence to support this has been obtained to date.

## 3 RAAS AND ITS RELATIONSHIP WITH COVID‐19: FRIEND OR FOE?

The identification of ACE2 as the receptor of SARS‐CoV‐2 places the RAAS at the centre of COVID‐19 pathogenesis. Furthermore, since this protease is involved in angiotensin peptide metabolism, which regulates the immune system, it is plausible to suggest that other components of the RAAS might play roles in COVID‐19 pathogenesis.

The RAAS is a regulatory proteolytic cascade involved in a variety of physiological functions in different organs, including the heart, kidneys, and lungs.^28^ The central axis involves the production of angiotensin II (Ang II) by proteolytic cleavage of angiotensin I (Ang I), promoted by angiotensin‐converting enzyme (ACE). Ang II elicits responses through two G protein‐coupled receptors (GPCR)—AT1 (AT1R) and AT2 (AT2R).^29^, ^30^ AT1R signaling is associated with effects that include vasoconstriction, a pro‐inflammatory response, and anti‐natriuresis.^29^, ^30^, ^31^ In contrast, AT2R signaling is reported to oppose AT1R effects, promoting vasodilation, an anti‐inflammatory response, and natriuresis.^29^, ^30^, ^32^, ^33^

ACE2 preferentially cleaves Ang II, producing Ang (1‐7)^34^, ^35^ and, to a lesser extent, cleaves Ang I to form Ang (1‐9), a peptide whose role remains unknown.^12^ The mechanisms mediating Ang (1‐7) effects involve a complex network mediated by another GPCR—the MAS receptor (MASR)—and AT2R.^13^, ^36^, ^37^, ^38^ Other peptides whose functions are less known, such as alamandine, might play a significant role in the RAAS, but their possible role in COVID‐19 will not be discussed in the present review.^39^, ^40^, ^41^

**FIGURE 2:**
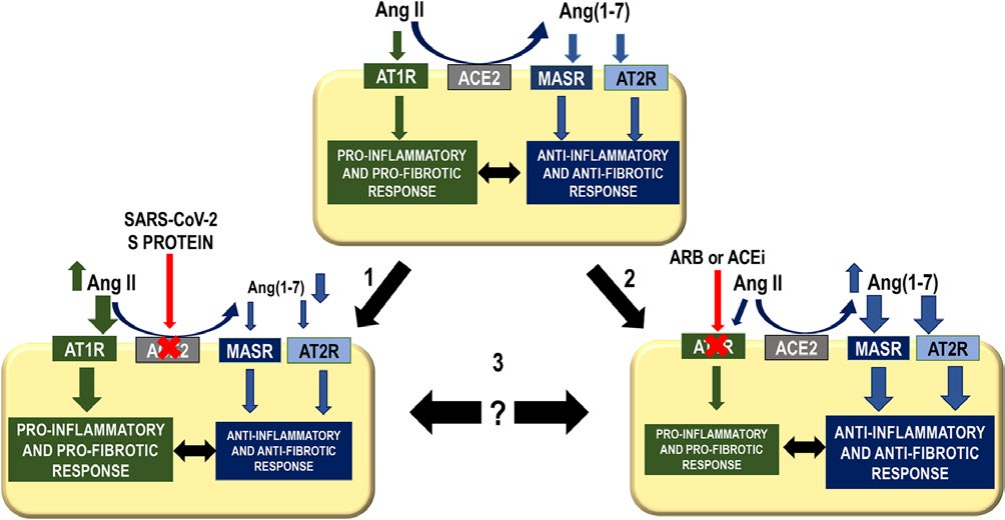
A fine balance between the ACE/Ang II/AT1R and ACE2/Ang (1‐7)/MASR axes of the renin–angiotensin–aldosterone system is observed in physiological conditions. (1) During SARS‐CoV‐2 infection, ACE2 is downregulated, promoting the ACE/Ang II/AT1R axis and, consequently, a proinflammatory and profibrotic response. (2) Patients with chronic diseases such as hypertension and diabetes are often treated with ARB/ACEi. These medications upregulate the ACE2/Ang (1‐7)/MASR axis, which in turn reduces inflammation and fibrosis signals. (3) How these different conditions are correlated, however, remains to be explored

There is now a consensus that the final effect of the RAAS cascade depends on a fine equilibrium between the ACE/Ang II/AT1R and ACE2/Ang (1‐7)/MASR/AT2R axes (Figure 2); their uncoupling is implicated in the pathogenesis of different lung diseases, including viral infection by respiratory syncytial virus and SARS‐CoV,^18^, ^42^ pulmonary hypertension,^43^, ^44^ and acute lung injury.^45^, ^46^ Accordingly, it has been previously shown that the SARS‐CoV S protein decreases ACE2 protein expression in the lung, increases Ang II levels, and enhances lung injury in an Ang II/AT1R‐dependent manner.^18^ These observations raise extremely relevant questions—is RAAS involved in the pathogenesis of COVID‐19? How? Is this a targetable therapeutic opportunity?

RAAS dysregulation is a well‐known mechanism involved in the genesis and progression of several comorbidities known to enhance COVID‐19 susceptibility, including hypertension and diabetes.^47^, ^48^, ^49^ Indeed, angiotensin AT1 receptor blockers (ARBs) and ACE inhibitors (ACEi) are the first‐line therapeutic strategies used to decrease cytokine production and halt end‐organ damage in both conditions.^50^, ^51^, ^52^, ^53^ These positive effects have been also observed after recombinant ACE2 treatment and Ang (1‐7) administration.^54^ These observations suggest the possibility of a therapeutic opportunity for the use of ARB and ACEi to attenuate patient responses to SARS‐CoV‐2 infection, in combination with viral‐targeted therapy.

On the other hand, some authors have observed that severe COVID‐19 cases were associated with chronic use of ARB and ACEi. One possible mechanism might be the upregulation of ACE2 expression induced by these treatments, as previously described,^55^, ^56^ although lung ACE2 expression in this setting has yet to be examined. This preliminary hypothesis led to a debate on whether ARB and ACEi should be discontinued in order to decrease COVID‐19 susceptibility.^57^ However, a consensus quickly emerged that these therapies should be maintained, since there is not enough evidence to support withdrawal of established therapeutic strategies against hypertension and diabetes (https://professional.heart.org/professional/ScienceNews/UCM_505836_HFSAACCAHA‐statement‐addresses‐concerns‐re‐using‐RAAS‐antagonists‐in‐COVID‐19.jsp,https://www.escardio.org/Councils/Council‐on‐Hypertension‐(CHT)/News/position‐statement‐of‐the‐esc‐council‐on‐hypertension‐on‐ace‐inhibitors‐and‐ang). This position seeks both to maintain comorbidities under control and to avoid secondary events during COVID‐19 infection. Corroborating this recommendation, Zhang et al found in a retrospective study that COVID‐19‐related mortality was significantly lower in patients treated with ARB/ACEi than in those not treated with these drugs.^58^

**FIGURE 3:**
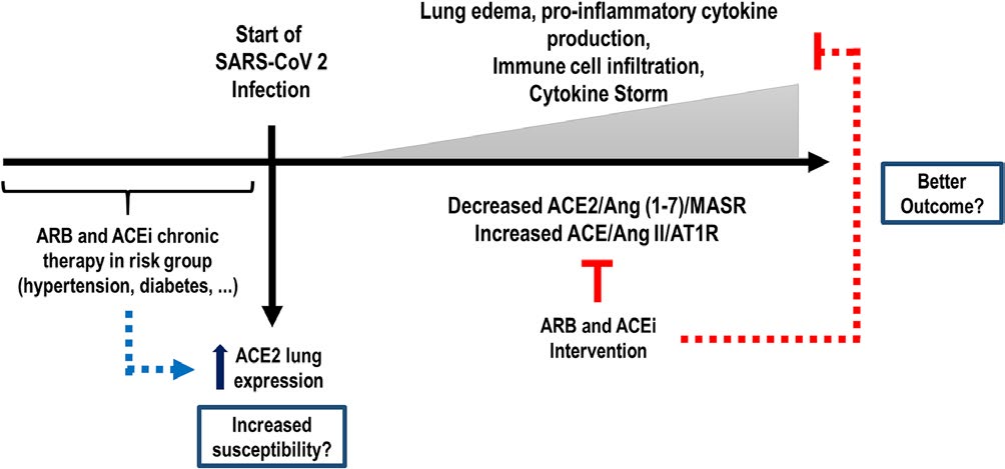
COVID‐19 disease pathogenesis and a proposal for ARB/ACEi‐based intervention. Schematic diagram of the time‐dependent correlation between RAAS activity and COVID‐19 pathogenesis. Risk‐group patients receiving ARB/ACEi likely overexpress ACE2, which could at least partly explain their higher susceptibility to severe illness. However, experimental evidence suggests that, during the course of SARS‐CoV‐2 infection, there is upregulation of the ACE/Ang II/AT1R axis to the detriment of the ACE2/Ang (1‐7)/MASR axis. This could account for the lung edema, immune cell infiltration within the lung, and cytokine storm observed in infected patients. In this stage of the disease, ARB/ACEi could represent a therapeutic opportunity to halt the host response to SARS‐CoV‐2 infection

Taken together, the evidence suggests a time‐dependent correlation between the RAAS and COVID‐19 pathogenesis (Figure 3). Initially, treatment with ARB and/or ACEi promotes an increase in ACE2/Ang (1‐7)/MASR axis activity, associated with an anti‐inflammatory profile. At this stage, SARS‐CoV‐2 binding and infection is promoted. As the course of infection progresses, the ACE2/Ang (1‐7)/MASR axis is downregulated, leading to internalization of ACE2 and a consequent reduction in its expression. This ACE2 downregulation enhances the ACE/Ang II/AT1R axis, which may explain several pathological events observed during COVID‐19 progression, such as lung edema, immune cell infiltration within the lung, and an intense proinflammatory response.

Importantly, vascular damage has been highlighted as a hallmark of COVID‐19 severity. Ackermann et al^59^ observed that marked endothelial injury, with signs of viral replication, accompanied by microangiopathy and thrombosis that are associated to severe COVID‐19. Indeed, it has been described that Ang II promotes vascular leakage, endothelial release of reactive oxygen species (ROS), and endothelial cell proliferation that is correlated with endothelial dysfunction.^60^, ^61^, ^62^, ^63^ Furthermore, ACE2 and TRMPS2 are expressed in endothelial cells, indicating possible productive replication of SARS‐CoV‐2 in this compartment.^64^ Based on these observations, it is plausible to postulate that SARS‐CoV‐2 infection and replication within lung microvasculature might contribute to initial increase in viral load. Therefore, increased Ang II/AT1R axis further promotes COVID‐19 progression to severe and fatal cases through, at least in part, induction of endothelium dysfunction and vascular permeability and, consequently, edema. The actual contribution of SARS‐CoV‐2 replication within the endothelial cells for COVID‐19 outcome should be further investigated. Other possible link between RAS and vascular damage could be the Kallikrein Kinin System. It is well known that ACE2 breaks down bradykinin into desArg9‐bradykinin, which, through B1 receptor activation, is involved in endothelial dysfunction.^65^

However, one fundamental question remains—why are hypertensive patients more susceptible to severe COVID‐19? One possibility comes from the observation that an overactivation of proinflammatory response triggered by SARS‐CoV‐2, the so‐called cytokine storm, is associated with worse outcomes.^2^, ^4^, ^66^ Huang et al showed that critically ill COVID‐19 patients have high levels of serum proinflammatory cytokines such as IL‐1β, IFN‐γ, IP‐10, and MCP‐1, which are associated with disease severity.^7^ Additionally, Qin et al showed that the number of helper, suppressor, and regulatory T cells is decreased.^67^ The cytokine storm leads to host cell damage, resulting in alveolar edema and pulmonary fibrosis as well as less‐known heart and kidney injuries.^28^

The evidence suggests that a low‐grade proinflammatory response could be implicated in hypertension having a causative relationship with changes observed in peripheral vascular resistance and the neural, cardiac, and renal systems.^68^ Ang II‐induced hypertension is known to involve polarization of CD4^+^ T cells toward the Th1 and Th17 phenotypes to the detriment of a Th2 phenotype, and involves an increase in IFN‐γ, IL‐6, and IL‐17.^69^, ^70^, ^71^ Furthermore, cytotoxic CD8^+^ T cells play an important role in this process, as their levels are increased in the kidney,^72^ a possible target organ of SARS‐CoV‐2. Interestingly, the innate immune response, which involves monocytes, macrophages, granulocytes, and dendritic cells, seems to be involved in endothelial dysfunction observed in Ang II‐induced hypertension.^68^

Based on these observations, we may infer that the association between hypertension and worse prognosis in COVID‐19 could be due to a preexisting proinflammatory state observed in hypertensive patients. Once a hypertensive patient is infected with SARS‐CoV‐2, sensitization of the immune system toward an overactivation of the inflammatory response could be associated with subsequent development of cytokine storm.

## 4 THERAPEUTIC OPTIONS FOR SARS‐COV‐2 TARGETING THE RAAS

The pathophysiological insights described above open up new possibilities for therapeutic proposals as follows: (a) use of protease inhibitors to block S protein priming. In this context, it has been proposed that the clinically approved serine protease inhibitor camostat mesylate might decrease SARS‐CoV‐2 entry in vitro.^11^ Clinical trials testing of the effect of camostat on COVID‐19 are now ongoing (NCT04321096); (b) use of chloroquine/hydroxychloroquine do inhibit viral replication, as demonstrated in different preclinical models.^73^, ^74^, ^75^ In the last months, the use of chloroquine/hydroxychloroquine has been extensively debated and further randomized, placebo‐controlled, clinical studies are required to clarify the benefits of chloroquine/hydroxychloroquine therapy for COVID‐19 patients. The purported molecular mechanism involves alkalization of endosomal pH and subsequent inhibition of membrane fusion^76^; (c) based on the homology between SARS‐CoV and SARS‐CoV‐2 S protein, antisera from immunized patients, ACE2 neutralizing antibody, or recombinant ACE2 have been proposed as possible inhibitors of viral engagement on epithelial cells.^11^, ^77^ Indeed, a monoclonal antibody against the receptor binding domain (RBD) of SARS‐CoV‐2, obtained from B cells of immunized COVID‐19 patients, has been shown to block the SARS‐CoV‐2 S protein/ACE2 interaction.^15^ Moreover, structural data on this interaction have highlighted design strategies for the development of molecular inhibitors ^21^, ^22^, ^23^, ^24^; d) based on the observation that ARBs and/or ACEi could blunt the proinflammatory response in different chronic degenerative diseases, it is plausible to suggest their use in severe cases of COVID‐19, where cytokine storm is one of the main concerns. Accordingly, Meng et al^78^ observed that Chinese hypertensive patients treated with ACEi or ARB who were stricken with COVID‐19 had a lower rate of progression to severe illness, with a trend toward lower IL‐6 plasma levels. A clinical trial of treatment with losartan is underway (NCT04328012).

## 5 CONCLUSION

COVID‐19 is spreading at an alarming rate worldwide. Although SARS‐CoV‐2 has structural and functional similarities with SARS‐CoV, new attributed capabilities of SARS‐CoV‐2 have been discovered. The absence of a vaccine or specific treatment and the intense immune response triggered by SARS‐CoV‐2 have been associated with the large number of infections and deaths. Here we highlighted a particularly important pathway—the renin–angiotensin–aldosterone system—whose role in COVID‐19 pathogenesis must be further understood in order to develop effective therapeutic strategies for COVID‐19.
